# Synthetic biology: the many facets of T7 RNA polymerase

**DOI:** 10.15252/msb.20145492

**Published:** 2014-07-30

**Authors:** David L Shis, Matthew R Bennett

**Affiliations:** Department of Biochemistry & Cell Biology, Rice UniversityHouston, TX, USA

## Abstract

Split T7 RNA polymerase provides new avenues for creating synthetic gene circuits that are decoupled from host regulatory processes—but how many times can this enzyme be split, yet retain function? New research by Voigt and colleagues (Segall-Shapiro *et al*, 2014) indicates that it may be more than you think.

Synthetic gene circuits have become an invaluable tool for studying the design principles of native gene networks and facilitating new biotechnologies (Way *et al*, 2014). Synthetic biologists often strive to build circuits within a framework that enables their consistent and robust operation across a range of hosts and conditions. Currently, however, each circuit must be fastidiously tuned and retuned in order to properly function within a particular host, leading to costly design cycles and esoteric conclusions. As a result, researchers have invested a great deal in developing strategies that decouple synthetic gene circuits from host metabolism and regulation. In their recent work, Segall-Shapiro *et al* (2014) address this problem by expanding the capabilities of orthogonal transcriptional systems in *Escherichia coli* using fragmented mutants of bacteriophage-T7 RNA polymerase (T7 RNAP).

T7 RNAP has had a long relationship with biotechnology and is renowned for its compactness and transcriptional activity. This single subunit polymerase strongly drives transcription from a miniscule 17-bp promoter that is orthogonally regulated in *E. coli*. In this context, orthogonal means that T7 RNAP will not transcribe genes driven by native *E. coli* promoters, and native polymerases in *E. coli* will not recognize T7 RNAP's special promoter—that is the two transcriptional systems leave each other alone. Interestingly, T7 RNAP drives transcription so strongly that, if left unregulated, it can quickly exhaust cellular resources and lead to cell death. Because of this, T7 RNAP has been leveraged in many situations calling for protein over-expression (Studier & Moffatt, 1986). Additionally, studies examining the binding of T7 RNAP to its promoter have identified a specificity loop within the enzyme that makes direct contact with the promoter between base pairs −11 and −8. This has led to a number of efforts that have generated T7 RNAP mutants with modified specificities to promoters orthogonal to the original (Chelliserrykattil *et al*, 2001).

Given the growing interest in the development of synthetic gene circuits, researchers have taken a renewed interest in T7 RNAP. The orthogonality, transcriptional activity and promoter malleability of T7 RNAP make the enzyme uniquely suited for use in synthetic gene circuits. Importantly, any modifications made to the enzyme increase the possible functionality of circuits. For instance, we recently utilized a split version of T7 RNAP in conjunction with promoter specificity mutants to create a library of transcriptional AND gates (Shis & Bennett, 2013). The split version of T7 RNAP was originally discovered during purification and shown to be active *in vitro* (Ikeda & Richardson, 1987). While the catalytic core and DNA-binding domain are both located on the C-terminal fragment of split T7 RNAP, the N-terminal fragment is needed for transcript elongation. Therefore, if the two halves of split T7 RNAP are placed behind two different inducible promoters, both inputs must be active in order to form a functional enzyme and activate a downstream gene. When the split mutant is combined with promoter specificity mutants, a library of transcriptional AND gates is created.

Segall-Shapiro *et al* take the idea of splitting T7 RNAP for novel regulatory architectures one step further. Instead of settling for the one split site already discovered, the authors first streamlined a transposon mutagenesis strategy (Segall-Shapiro *et al*, 2011) to identify four novel cut sites within T7 RNAP. By expressing T7 RNAP split at two different sites, they create a tripartite T7 RNAP—a polymerase that requires all three subunits for activity. The authors suggestively designate the fragments of the tripartite enzyme as ‘core’, ‘alpha’, and ‘sigma’ (Fig1) and they go on to show that tripartite T7 RNAP can not only be used to create 3-input AND gates, but it also works as a ‘resource allocator’. In other words, the transcriptional activity of the split polymerase can be regulated by limiting the availability of core and/or alpha fragment, or by expressing additional sigma fragments. The authors demonstrate strategies to account for common pitfalls in synthetic gene networks such as host toxicity and plasmid copy number variability.

**Figure 1:**
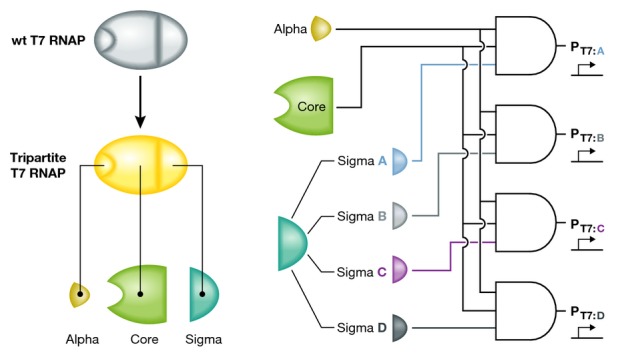
Segall-Shapiro *et al* extend previous efforts to engineer split T7 RNAP by fragmenting the enzyme at two novel locations to create a tripartite transcription complex Co-expressing different sigma fragments with the alpha and core fragments enables a network of multi-input transcriptional AND gates.

The tripartite T7 RNAP presented by Segall-Shapiro *et al* expands the utility of T7 RNAP in orthogonal gene circuits. Until now, while T7 RNAP has been attractive for use in synthetic gene circuits, the inability to regulate its activity has often prevented its use. Splitting the protein into fragments and regulating the transcription complex by fragment availability brings the regulation of T7 RNAP closer to the regulation of multi-subunit prokaryotic RNA polymerases. Sigma fragments direct the activity of the transcription complex much like σ-factors, and the alpha fragment helps activate transcription in the same way as α-fragments of prokaryotic polymerases. For additional regulation, the authors note that the tripartite T7 RNAP can be further split at the previously discovered split site to create a four-fragment enzyme.

More nuanced regulation using split T7 RNAP may be possible with the addition of heterodimerization domains that can drive the specific association of fragments. This strategy has been successfully applied to engineer specificity and signal diversity in two-component signaling pathways (Whitaker *et al*, 2012). The activity of T7 RNAP might also be directed to various promoters by using multiple sigma fragments simultaneously, just as σ-factors do in *E. coli*. Finally, synthetic gene circuits driven primarily by T7 RNAP create the possibility of easily transplantable gene circuits. A synthetic gene circuit driven entirely by fragmented T7 RNAP would depend more on fragment availability than unknown interactions with host metabolism. This would enable rapid prototyping of synthetic gene circuits in laboratory-friendly strains or cell-free systems (Shin & Noireaux, 2012) before transplantation into the desired host.
